# Reducing Bias in the Evaluation of Robotic Surgery for Lung Cancer Through Machine Learning

**DOI:** 10.3390/cancers17203347

**Published:** 2025-10-17

**Authors:** Alain Bernard, Jonathan Cottenet, Pascale Tubert-Bitter, Catherine Quantin

**Affiliations:** 1 Department of Thoracic and Cardiovascular Surgery, University Hospital, 21000 Dijon, France; alain.bernard@chu-dijon.fr; 2 Department of Biostatistics and Bioinformatics, University Hospital, 21000 Dijon, France; jonathan.cottenet@chu-dijon.fr; 3 CESP, Inserm, UVSQ, High-Dimensional Biostatistics for Drug Safety and Genomics, Paris-Saclay University, 94807 Villejuif, France; pascale.tubert@inserm.fr; 4 Institut National de la Santé et de la Recherche Médicale, Centre Investigation Clinique 1432, 21000 Dijon, France

**Keywords:** robotic surgery, lung cancer, machine learning, propensity score, 90-day mortality

## Abstract

**Simple Summary:**

Conventional methods such as logistic regression for propensity score estimation often failed to adequately balance covariates, reducing the reliability of comparisons between robot-assisted surgery (RAS) and thoracotomy. Machine learning had been proposed as a potential solution, but its application in this context remained underexplored. This study demonstrates that machine learning models, particularly XGBoost and Random Forest, outperform traditional logistic regression in estimating propensity scores and reducing selection bias. By achieving better discrimination and enhanced covariate balance, these models provide more robust comparative analyses, with a significant reduction in 90-day mortality compared to thoracotomy. The findings support the integration of machine learning in surgical outcomes research, particularly for improving causal inference in observational studies. Clinically, the results reinforce the benefits of RAS, which may influence surgical decision-making and patient selection criteria. Policymakers may also consider these findings when assessing the adoption of robotic surgery and its impact on healthcare outcomes.

**Abstract:**

Background: Robot-assisted surgery (RAS) is a major innovation in the treatment of lung cancer, offering advantages in surgical precision and reducing postoperative complications. However, its impact on 90-day mortality remains controversial due to methodological biases in comparative studies. This study uses machine learning methods to improve propensity score estimation and reduce selection bias. Methods: We used the French national hospital database (PMSI) to identify patients who underwent lung resection for cancer between 2019 and 2023. Four models were applied for propensity score estimation: logistic regression, Random Forest, Gradient Boosting Machine (GBM), and XGBoost. Group balancing was achieved through propensity score weighting and matching, followed by logistic regression analysis to estimate the effect of RAS on 90-day mortality. Results: Among the 30,988 patients included, 5717 (18.5%) underwent robot-assisted surgery, while 25,271 (81.5%) underwent thoracotomy. RAS patients had a lower prevalence of comorbidities and earlier-stage tumors. XGBoost was the most effective model for propensity score estimation, with an AUC ROC of 0.9984 and a Brier Score of 0.0119. The adjusted analysis showed a significant reduction in 90-day mortality in the RAS group (OR = 0.39, 95% CI: 0.34–0.45) with weighting and (OR = 0.58, 95% CI: 0.48–0.70) with matching. Conclusions: The application of machine learning to adjust for selection bias allowed for better control of confounding factors in the analysis of the effect of RAS on 90-day mortality. Our results suggest a potential benefit of robotic surgery compared to thoracotomy, although further studies are needed to confirm these findings.

## 1. Introduction

Robot-assisted surgery (RAS) represents a major advancement in the management of lung cancer, offering improved visualization, greater surgical precision, and a reduction in postoperative complications compared to traditional thoracotomy. Several studies have shown that RAS may lead to reduced blood loss, shorter hospital stays, and faster recovery [1,2]. However, despite these technical and clinical benefits, its impact on 90-day mortality remains controversial due to methodological weaknesses in comparative studies.

One meta-analysis [3] has highlighted that observational studies evaluating the effectiveness of RAS often suffer from selection bias, a lack of rigorous control over covariates, and heterogeneity in the studied populations. These limitations make it difficult to generalize the findings and pose a challenge in the impartial evaluation of RAS compared to thoracotomy. Furthermore, conventional propensity score methods, which are widely used to adjust for differences between groups, do not always sufficiently reduce bias in observational studies [4].

To address these limitations, we propose leveraging advancements in artificial intelligence (AI) and employing multiple machine learning (ML) methods to improve propensity score estimation and reduce bias related to patient selection [5,6,7,8]. The objective of this study is to use ML to compare the impact of robot-assisted surgery versus thoracotomy on 90-day mortality while minimizing methodological biases. To achieve this, we applied the following algorithms to estimate a propensity score and attempt to mimic a randomized controlled trial: random forest (RF), gradient boosting machine (GBM), XGBoost (XGB), and logistic regression (LR).

By combining these approaches, we aimed to achieve a better estimation of the propensity score and identify the most effective model for reducing bias, ensuring a more reliable and robust comparison between RAS and thoracotomy.

## 2. Materials and Methods

### 2.1. Database and Inclusion

This study utilized the French national hospital database from the Programme de Médicalisation des Systèmes d’Information (PMSI), which includes discharge abstracts for all inpatient admissions to both public and private hospitals across France. Diagnoses recorded during the hospital stay are classified according to the 10th edition of the International Classification of Diseases (ICD-10) [9,10], while medical and surgical procedures performed during hospitalization are coded according to the French Common Classification of Medical Procedures (CCAM). We included patients from this database who underwent pulmonary resection for lung cancer (LC) in France between 2019 and 2023. Specifically, we selected patients with a primary diagnosis of LC (ICD-10 code C34) along with a corresponding surgical procedure for LC (CCAM codes) performed during the same hospital admission [11,12].

### 2.2. Patient Characteristics

For each patient, we collected data on age, gender, and surgery-related factors, including the type of surgical approach and the nature of the resection. Additionally, we considered comorbidities (pulmonary, cardiovascular, peripheral vascular, liver, neurological, kidney, hematologic, metabolic, infectious), cerebrovascular events, anemia, and therapies. We also calculated the modified Charlson Comorbidity Index (CCI) to assess the overall comorbidity burden [13]. The year variable was transformed into dummy variables, with 2019 as the reference year.

To enhance the analysis, we incorporated variables from the scientific literature, including body mass index (BMI), forced expiratory volume in one second (FEV1), World Health Organization (WHO) performance status, histological type (adenocarcinoma), and tumor stage (T and N). Values were extracted from a literature review [4]. The weighted mean and standard deviation were estimated for age, BMI, and FEV1, while percentages were estimated for binary and categorical variables.

To enhance the robustness of the analyses, simulations were conducted by generating 1000 samples for robot-assisted surgery and 1000 samples for thoracotomy, resulting in a final dataset of 2000 observations. This approach aimed to strengthen the statistical power of the study and reduce potential biases associated with small sample sizes. To ensure consistency and homogeneity between the compared groups, the simulated dataset was merged with the PMSI database using surgical approach, age, and sex as matching variables. This matching process helped to align the simulated data with real-world patient characteristics, thereby enhancing the validity of the comparative analysis.

The primary outcome measure used in this study was 90-day mortality, which included deaths occurring during the same hospitalization as the lung cancer surgery or deaths occurring in another facility within 90 days of the procedure.

### 2.3. Modeling and Propensity Score Estimation

Propensity score estimation is a statistical approach used to adjust for differences between groups in observational studies, thereby reducing selection bias [14]. This score represents the conditional probability of a patient receiving a given treatment based on observed characteristics. In this study, we employed four methods for propensity score estimation:

- Logistic Regression (LR): A classical statistical model for propensity score estimation, based on a logistic function that models the probability of belonging to a treatment group according to covariates [8].
- Random Forest (RF): A supervised learning algorithm based on an ensemble of decision trees. Each tree is trained on a subsample of the data, and the final prediction is obtained through majority voting. RF is robust to nonlinear interactions and highly correlated variables [5]. To mitigate the risk of overfitting (i.e., when a model learns the training data excessively well but fails to generalize to unseen data) and to prevent data leakage (the inadvertent use of future or test set information during training), we implemented a rigorous methodological framework. The Random Forest algorithm, which aggregates multiple decision trees built from random subsamples of the dataset, is widely recognized for its robustness in handling correlated variables and complex interactions. Model hyperparameters (including the number of trees, tree depth, and the minimum number of patients per terminal node) were optimized through a systematic grid search embedded within a 5-fold cross-validation procedure. In this approach, the dataset was partitioned into five subsets of comparable size: in each iteration, four subsets were used for training and one for validation, with roles rotated to ensure that all data contributed to both training and validation. Final performance metrics were derived exclusively from an independent test set, which was set aside at the outset and never used during model training or parameter tuning. This strategy ensures that the reported results reflect genuine generalizability rather than methodological artifacts.
- Gradient Boosting Machine (GBM): A supervised learning method that progressively improves model performance by minimizing a loss function. Each new tree corrects the errors of previous trees, allowing for higher predictive accuracy [6]. The Gradient Boosting Machine (GBM) is a supervised statistical learning method that combines multiple weak learners (decision trees) to predict a clinical outcome. Each successive tree is built to correct the errors of the previous one, thereby progressively improving predictive accuracy.

- Learning rate: regulates the contribution of each new tree; excessively high values may lead to model instability, while overly low values may slow down learning.
- n_estimators: total number of trees constructed; larger values allow the model to capture more complex relationships, but increase the risk of overfitting.
- max_depth: maximum depth of the trees; this parameter constrains individual tree complexity to prevent the model from fitting only the idiosyncrasies of the training sample.
- subsample: proportion of the data used at each iteration; this introduces randomness that is beneficial in reducing the risk of overfitting.

Optimal hyperparameters were identified through a systematic grid search, testing different parameter combinations. The performance of each combination was assessed using 5-fold cross-validation: the dataset was divided into five equally sized subsets, with the model trained on four-fifths and tested on the remaining fifth, rotating subsets across five iterations. This approach ensures that results are robust and generalizable, while minimizing the risk of developing a model overly tailored to the training data.

- XGBoost (XGB): An optimized variant of GBM that incorporates regularization to prevent overfitting and improve computational efficiency. This algorithm is well known for its high performance and fast learning capabilities [7]. XGBoost (Extreme Gradient Boosting) is an optimized implementation of the Gradient Boosting Machine (GBM). It is widely recognized for its high predictive performance and computational efficiency. The algorithm incorporates regularization mechanisms to reduce the risk of overfitting, i.e., excessive adaptation to the training data.

- learning_rate: controls the contribution of each new tree; lower values lead to a more gradual and stable learning process.
- n_estimators: total number of trees constructed; directly influences the complexity and accuracy of the model.
- max_depth: maximum depth of the trees; constrains tree complexity to avoid oversensitivity to sample-specific patterns.
- alpha and lambda: regularization parameters (penalties) that regulate model complexity and mitigate overfitting.
- subsample and colsample_bytree: proportions of data and variables used at each iteration, introducing beneficial randomness that enhances robustness.

Hyperparameter optimization was performed using a Bayesian optimization approach, which efficiently explores the parameter space to identify combinations that maximize predictive accuracy while limiting overfitting. Model performance was evaluated using 10-fold cross-validation, whereby the dataset was partitioned into ten subsets: in each iteration, the model was trained on nine-tenths and tested on the remaining tenth, repeated across all partitions. This strategy ensures reliable and generalizable results.

The application of these different methods allowed us to assess their respective ability to balance covariates and improve comparability between groups by selecting the model that provided the best reduction in bias.

To evaluate the performance of the ML models in propensity score estimation, we used several standard metrics: Area Under the Curve (AUC) ROC [15], Accuracy [16] and Calibration (Brier Score) [17].

To ensure robust model performance evaluation, we applied cross-validation techniques to assess model discrimination and calibration. Standardized Mean Differences (SMD) were used to evaluate covariate balance before and after adjustment. An SMD value below 0.1 is generally considered indicative of good post-adjustment balance [18].

The final model selection was based on its ability to optimize bias reduction and maximize accuracy, ensuring the most reliable propensity score estimation for subsequent analysis.

### 2.4. Bias Reduction Strategy

To minimize selection bias and ensure optimal comparability between study groups, we applied two propensity score-based approaches:

- Propensity score weighting assigns a weight to each observation to balance the distribution of covariates between treatment groups, thereby minimizing initial imbalances [19].
- Propensity score matching: Matches patients who underwent robot-assisted surgery with thoracotomy patients who have similar characteristics, reducing potential confounding sources [20].

To assess the influence of surgical approach on 90-day mortality, we applied an LR model after adjustment using propensity score weighting and matching. This approach allowed us to estimate the odds ratio (OR) and its 95% confidence interval (CI), providing a robust measure of the association between surgical technique and postoperative mortality [8].

The various steps mentioned above are summarized in Figure S1. All analyses were performed using R software version 4.4.1.

## 3. Results

### 3.1. Patient and Hospital Characteristics by Surgical Approach

The analysis of patient and healthcare facility characteristics according to the surgical approach revealed significant differences between the groups undergoing robot-assisted surgery (RAS) and those undergoing thoracotomy. Among the 30,988 patients included in the study, 5717 (18.5%) underwent robot-assisted surgery, while 25,271 (81.5%) underwent thoracotomy (Table S1), with an associated 90-day mortality of 3.1% for patients who underwent robot-assisted, and 7.8% for patients who underwent thoracotomy (*p* < 0.001).

Regarding comorbidities, patients who underwent robotic surgery had a lower prevalence of cardiovascular diseases (15.2% vs. 17.9%, *p* < 0.001), peripheral vascular diseases (8.8% vs. 11.1%, *p* < 0.001), and hematologic disorders (2.3% vs. 5.7%, *p* < 0.001) compared to the thoracotomy group. Conversely, the proportion of patients with adenocarcinoma was significantly higher in the robotic surgery group (77.3% vs. 63.2%, *p* < 0.001), as was the frequency of T1-stage tumors (71.4% vs. 38.3%, *p* < 0.001), suggesting a selection bias favoring smaller tumors.

In terms of respiratory function and general health status, patients in the robotic surgery group had significantly higher forced expiratory volume (FEV1) (79.4% vs. 73.9%, *p* < 0.001) and a higher body mass index (BMI) (26.8 kg/m^2^ vs. 25.4 kg/m^2^, *p* < 0.001). Additionally, a greater proportion of patients with a WHO performance status of 0 was observed in the robotic surgery group (61.2% vs. 41.1%, *p* < 0.001), indicating better preoperative functional status.

Regarding surgical technique, lobectomies accounted for 97.2% of procedures in the robotic surgery group compared to 65.0% in the thoracotomy group (*p* < 0.001), while pneumonectomies and extended resections were significantly less frequent in the robotic group (0.3% vs. 8.3% and 0.1% vs. 17.8%, respectively; *p* < 0.001), suggesting a selection of patients for less complex procedures.

Finally, the analysis of hospital characteristics revealed that robotic surgery was more frequently performed in academic hospitals (48.7% vs. 48.1%, *p* < 0.001) and in high-volume surgical centers (266.9 vs. 253.1 annual interventions on average, *p* < 0.001). A significant temporal trend was observed, with a progressive increase in robotic surgery use, reaching 41.8% of interventions in 2023 compared to 3.1% in 2019 (*p* < 0.001).

These results suggest that robotic surgery is preferentially performed on patients with better general health, preserved pulmonary function, early-stage tumors, and requiring less invasive surgical procedures, reflecting a selection bias favoring less complex cases in this population.

### 3.2. Model Performance Evaluation

The performance evaluation of the models is presented in Table 1. In terms of discrimination, measured by the area under the ROC curve (AUC ROC), the RF model (AUC = 0.9987) and XGBoost (AUC = 0.9984) achieved the highest values, indicating an excellent ability to distinguish between the studied groups. Regarding overall accuracy, XGBoost (98.67%) outperformed the other models, followed by RF (98.38%) and GBM (98.14%). In contrast, LR showed significantly lower performance (AUC = 0.9162, accuracy = 87.44%). These findings confirm the superiority of non-parametric ML models over LR for propensity score estimation.

In terms of predictive probability calibration, the Brier Score was the lowest for XGBoost (0.0119), followed by RF (0.0121) and GBM (0.0165), further confirming the robustness of XGBoost’s predictions and its ability to minimize the mean squared error between estimated probabilities and actual observations.

Before matching or weighting, the median SMD was 0.142 (Table S2). The analysis of SMD, summarized in Table 2, assesses the ability of different adjustment methods to balance covariates between groups. The medians and extreme values of the SMDs were lower with XGBoost and RF, suggesting better group homogeneity after adjustment. Furthermore, propensity score weighting proved to be more effective than matching, with SMD values closer to zero, indicating a greater reduction in selection bias. Furthermore, the number of patients was reduced after matching, with 5717 patients in the thoracotomy group and 5717 patients in the robot-assisted surgery group.

**Figure 1:**
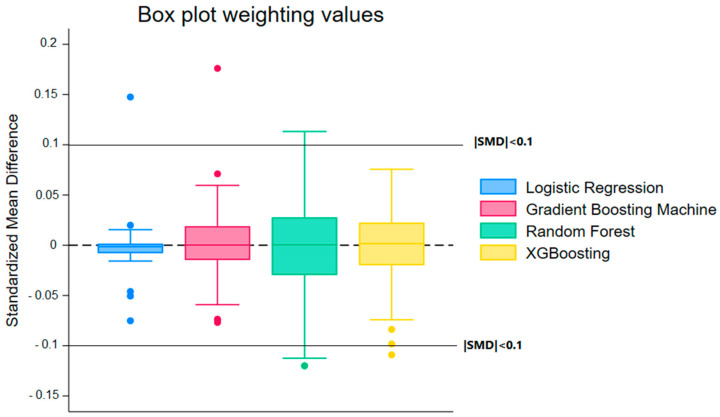
Distribution of Standardized Mean Differences (SMD) After Weighting for Different Machine Learning Models. The figure illustrates the balance of covariates between the compared groups, with SMD values close to zero indicating better adjustment. The models include Random Forest, Logistic Regression, Gradient Boosting Machine, and XGBoost. The median, quartiles, and extreme values are represented for each model, highlighting the superior performance of machine learning-based methods, particularly XGBoost, in minimizing selection bias.

**Figure 2:**
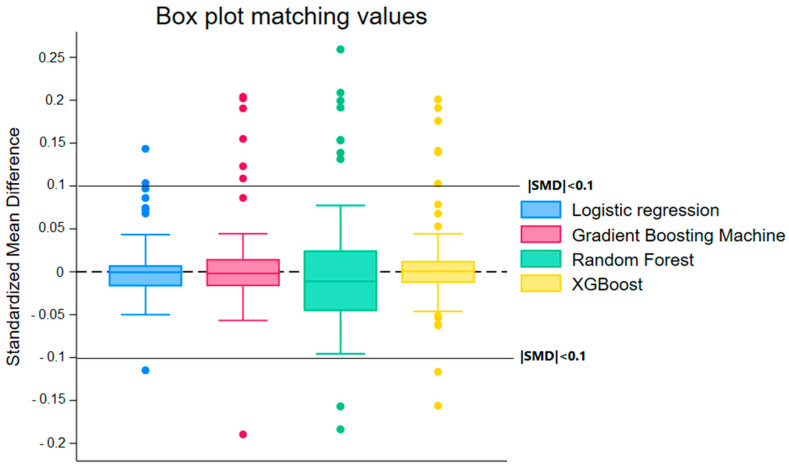
Distribution of Standardized Mean Differences (SMD) After Propensity Score Matching for Different Machine Learning Models. The figure compares the performance of Random Forest, Logistic Regression, Gradient Boosting Machine, and XGBoost in balancing covariates between groups. SMD values close to zero indicate better balance. The boxplots show that while all methods improve covariate balance, the dispersion of SMDs is greater after matching than after weighting, with more frequent extreme values for certain models, particularly Random Forest.

Figure 1 and Figure 2, depicting the distributions of SMDs after weighting and matching, respectively, support these findings. Figure 1 shows that XGBoost and RF generate distributions tightly centered around zero, demonstrating significant improvement in covariate balance after weighting.

Conversely, Figure 2, illustrating the effects of matching, shows that this method also improves group balance but with greater dispersion in SMD values, indicating relatively lower effectiveness compared to weighting.

Overall, these analyses indicate that XGBoost is the best-performing model for propensity score estimation, given its superior performance in discrimination (AUC ROC), accuracy, and calibration (Brier Score). Regarding bias reduction, propensity score weighting emerged as the most effective approach, minimizing differences between the compared groups more efficiently than matching.

These results suggest that the combination of XGBoost for propensity score estimation and propensity score weighting constitutes the optimal strategy to ensure a rigorous and robust comparison between robot-assisted surgery and thoracotomy in the treatment of lung cancer.

### 3.3. Effect of Robotic Surgery on 90-Day Mortality

In this study, we adopted two distinct approaches to estimate the effect of robot-assisted surgery (RAS) compared to thoracotomy on 90-day mortality.

The OR and its 95% CI were estimated using propensity score weighting based on the XGBoost model. The results indicate that robotic surgery is associated with a statistically significant reduction in the risk of 90-day mortality (OR = 0.39 [0.34–0.45]), suggesting a decreased risk of death compared to thoracotomy.

Additionally, a propensity score matching approach, followed by LR modeling, was applied. This method provided an adjusted OR estimate of 0.58 [0.48–0.70], also confirming a reduction in mortality risk associated with robotic surgery. The absolute risk reduction and its 95% CI were 2.66% [1.88–3.44], and the number needed to treat (NNT) and its 95% CI were 38 [29–53]; 38 patients need to be treated with RAS to prevent one death at 90 days.

These findings suggest that propensity score weighting using XGBoost is the most robust method for estimating the causal effect of robot-assisted surgery on 90-day mortality, as it more effectively reduces selection bias inherent to observational studies. The matching method with LR supports this trend, although its estimates exhibit slightly greater variability.

## 4. Discussion

### 4.1. Key Findings

The results of this study confirm the value of ML in evaluating robot-assisted surgery (RAS) for the treatment of lung cancer. By leveraging different ML approaches to estimate the propensity score, we were able to minimize selection bias and enhance the robustness of comparisons between RAS and thoracotomy.

Although LR is widely used for propensity score estimation, it requires assumptions regarding variable selection, their distributions, and the absence of interactions. ML algorithms, such as RFs, regression trees, neural networks, and gradient boosting, offer a promising alternative by capturing non-linear relationships and handling high-dimensional data. ML can potentially improve propensity score estimation by better accounting for interactions between variables [21,22,23].

### 4.2. Comparison with Existing Literature

Currently, few studies use ML models to estimate propensity scores. A recent literature review identified seven studies that utilized ML models [24]. The ML algorithms used included RFs, causal forests, Bayesian additive regression trees, neural networks, gradient boosting, and boosted regression trees. However, most studies do not report or display the performance metrics of the selected ML models. They often fail to mention the hyperparameter selection process, strategies to avoid overfitting, or the evaluation of performance metrics, limiting critical assessment and comparison with parametric approaches.

### 4.3. Bias Reduction and Model Performance

The analysis of ML model performance highlighted the superiority of XGBoost and RF compared to classical logistic regression. These models demonstrated better discrimination (AUC > 0.99) and optimized calibration (lower Brier Score). These results align with the findings of Chen and Guestrin [7] regarding XGBoost’s efficiency in reducing estimation bias and Breiman [5] on the robustness of RF in handling complex datasets.

The use of propensity score weighting allowed for a more effective homogenization of the compared groups than matching, as confirmed by the lower Standardized Mean Differences (SMD). Austin and Stuart [19] showed that weighting improves group comparability in observational studies, thereby reducing the risk of confounding.

Recent research has increasingly focused on the application of ML techniques and propensity score matching in the context of lung cancer. These methodologies aim to enhance the accuracy of predictions regarding patient outcomes and treatment efficacy while addressing confounding variables that may bias results. One significant aspect of this research is the use of propensity score matching to control for confounding factors in clinical studies. For instance, Wu et al. conducted a propensity score matching analysis to evaluate the impact of primary tumor surgery on survival rates in cancer patients with synchronous solitary bone metastasis. They demonstrated the effectiveness of propensity score matching in adjusting for baseline confounding variables [25]. Similarly, Mansur et al. employed propensity scores to match patients with primary clear cell adenocarcinoma of the lung and those with lung adenocarcinoma, highlighting the utility of LR models in calculating these scores [26]. This approach is crucial in ensuring that comparisons between treatment groups are valid and that the observed outcomes can be attributed to the interventions rather than confounding factors. Moreover, ML models have shown promise in classifying lung cancer subtypes, particularly feature engineering approaches, which yielded high accuracy rates in identifying various lung cancer classes, with the XGBoost model achieving an impressive accuracy of 99% [27], which is vital for personalized medicine. The integration of propensity score matching with ML techniques is particularly noteworthy. For example, Yang et al. utilized propensity scores to analyze the effects of mediastinal lymph node dissection on survival in patients undergoing colorectal cancer-related pulmonary metastasectomy. Their findings indicated that while certain factors adversely affected survival, the role of lymph node dissection remained ambiguous, underscoring the complexity of treatment outcomes in lung cancer [28,29]. This interplay between statistical methods and ML can enhance the robustness of findings in lung cancer research, allowing for more nuanced insights into treatment efficacy. In summary, the combination of propensity score matching and ML represents a significant advancement in lung cancer research. These methodologies not only help in controlling for confounding variables but also improve the predictive accuracy of models used to classify and treat lung cancer. As research continues to evolve, the integration of these approaches will likely lead to more effective and personalized treatment strategies for lung cancer patients.

### 4.4. Impact of Robot-Assisted Surgery on 90-Day Mortality

Adjusted analyses indicate a significant reduction in 90-day mortality among patients undergoing RAS, with an adjusted OR of 0.39 [0.34–0.45] after weighting and 0.58 [0.48–0.70] after matching. These results suggest that RAS is associated with a lower risk of postoperative death. We are surprised by the significant reduction in the risk of death at 90 days. This result is similar to the meta-analysis by KE O’Sullivan [30], which reports an OR of 0.53 [0.33–0.85] compared to thoracotomy. We used thoracotomy as the control, which may be debatable since many studies consider VATS as the reference surgical approach. However, the primary objective of our study was to compare machine learning models to logistic regression, which is conventionally used for propensity score estimation. Once machine learning models are validated, a study comparing robotic surgery to VATS will become essential.

### 4.5. Limitations

First, it is important to note that the selection of RAS patients introduces potential biases, particularly a higher proportion of early-stage tumors and patients with better preoperative conditions.

The quality of coding information in the hospital database may also be called into question, with the risk of underestimating certain comorbidities. In France, the quality of comorbidity coding has significantly improved in recent years, particularly due to its impact on hospital funding. Additionally, a national external quality assessment program has been added to each hospital’s internal quality assessment to check the quality of discharge abstracts.

The PMSI database lacks detailed information on certain prognostic factors, such as postoperative pulmonary function and complications specific to the surgical technique. Our study has the usual limitations of medico-administrative studies, including the absence of variables such as TNM stage or FEV1. For this reason, we enriched the PMSI database with TNM, histology, FEV1, BMI, and WHO variables based on literature data. However, these results should be interpreted with caution, given the potential risk of bias; the approach used risks introducing artificial balance rather than true patient-level covariates.

Although ML improves covariate adjustment, it does not replace a randomized controlled trial, which remains the gold standard for assessing causal effects. The rapid evolution of robotic surgery technologies could influence long-term outcomes. Future studies incorporating updated data and more recent cohorts will help refine these analyses. Moreover, the main drawback of ML models is their “black box” nature, which can make etiological interpretations difficult. Caution should always be exercised when interpreting the causal implications of real-world studies, due to the inherent biases of observational studies.

### 4.6. Strength

This study’s strength lies in its use of advanced ML techniques to improve propensity score estimation and reduce selection bias in evaluating robot-assisted surgery (RAS) for lung cancer. By applying models like XGBoost and RF, the study achieves superior discrimination (AUC > 0.99) and better covariate balance than traditional logistic regression. The large-scale national database (PMSI) enhances the study’s generalizability, while the rigorous bias reduction approach strengthens the reliability of findings. These results provide valuable evidence supporting the benefits of RAS in reducing 90-day mortality.

### 4.7. Unanswered Questions and Future Research

Despite these methodological advancements, ML does not replace the need for randomized controlled trials, which remain the gold standard for assessing causal effects. Thus, comparisons with VATS, which is now more common than thoracotomy, will be essential in future work. These future studies should incorporate updated data, larger cohorts, and direct comparisons with VATS to further validate these findings. We therefore plan, in future work, to compare RAS with two surgical approaches (thoracotomy and VATS), using more recent medico-administrative data. Furthermore, efforts to improve the interpretability of machine learning models will be essential to facilitate their use in clinical decision-making and, in particular, their effect on patient selection criteria. The investigation of interpretable machine learning models (e.g., SHAP values) would also be useful for bolstering confidence in study findings. These robust findings could then be translated into strategies for improving surgical training.

## 5. Conclusions

This study highlights the value of machine learning in refining the assessment of robot-assisted surgery (RAS) for lung cancer by improving propensity score estimation and reducing selection bias. It also demonstrates the innovation of employing machine learning. By leveraging advanced algorithms such as XGBoost and Random Forest, we demonstrated superior model performance compared to traditional logistic regression, achieving better discrimination and covariate balance. Our results suggest that RAS is associated with a reduction in 90-day mortality compared to thoracotomy. This association should be confirmed by further studies, given the biases inherent in observational studies and pending robust studies comparing RAS to VATS.

Overall, this study underscores the importance of using advanced analytical methodologies in surgical outcomes research and highlights the potential of artificial intelligence to improve evidence-based decision-making in thoracic oncology.

## Figures and Tables

**Table 1:** Model Performance.

Model	AUC ROC	Accuracy	Brier Score
Random Forest (RF)	0.9987	0.9838	0.0121
Logistic Regression (LR)	0.9162	0.8744	0.0870
Gradient Boosting Machine (GBM)	0.9954	0.9814	0.0165
XGBoost (XGB)	0.9984	0.9867	0.0119

**Table 2:** Statistics of Standardized Mean Differences (SMD).

Model	Median (Weighting)	Min (Weighting)	Max (Weighting)	Median (Matching)	Min (Matching)	Max (Matching)
Random Forest (RF)	0.0004	−0.12	0.1132	−0.0112	−0.1837	0.2592
Logistic Regression (LR)	−0.0014	−0.0752	0.1475	−0.0005	−0.1149	0.1434
Gradient Boosting Machine (GBM)	0.0002	−0.0769	0.176	−0.0019	−0.1896	0.204
XGBoost (XGB)	0.0016	−0.1091	0.0756	0.0005	−0.1562	0.2009

## Data Availability

The use of these data by our department was approved by the National Committee for data protection. We are not allowed to transmit these data. PMSI data are available for researchers who meet the criteria for access to these French confidential data (this access is submitted to the approval of the National Committee for data protection) from the national agency for the management of hospitalization (ATIH-Agence technique de l’information sur l’hospitalisation). Address: Agence technique de l’information sur l’hospitalisation, 117 boulevard Marius Vivier Merle—69329 Lyon Cedex 03.

## Supplementary Materials

The following supporting information can be downloaded at: https://www.mdpi.com/article/10.3390/cancers17203347/s1, Table S1: Patients and hospitals characteristics according to approach; Table S2: Variables used in propensity and Standardized Mean Difference (SMD) before matching and weighting; Figure S1: Modeling pipeline.

## Abbreviations

The following abbreviations are used in this manuscript:

AUC	Area Under the Curve
AI	artificial intelligence
BMI	body mass index
CCI	Charlson Comorbidity Index
CI	confidence interval
FEV1	forced expiratory volume in one second
CCAM	French Common Classification of Medical Procedures
PMSI	French national hospital database
GBM	Gradient Boosting Machine
ICD-10	International Classification of Diseases
LR	logistic regression
LC	lung cancer
ML	machine learning
OR	odds ratio
RF	random forest
RAS	Robot-assisted surgery
SMD	Standardized Mean Differences
VATS	video-assisted thoracic surgery
WHO	World Health Organization
XGB	XGBoost
