# Supplementary material for: Reducing Bias in the Evaluation of Robotic Surgery for Lung Cancer Through Machine Learning
Source: Cancers (Basel). 2025 Oct 17;17(20):3347. doi: 10.3390/cancers17203347 (PMC12563986; doi:10.3390/cancers17203347)

Table S1. Patients and hospitals characteristics according to approach

	Thoracotomy 25,271	Robot assisted surgery 5,717	p-value
Pulmonary disease			
0	16,382 (64.8%)	4,147 (72.5%)	<0.001
1	8,889 (35.2%)	1,570 (27.5%)	
Heart disease			
0	20,738 (82.1%)	4,850 (84.8%)	<0.001
1	4,533 (17.9%)	867 (15.2%)	
Peripheral vascular disease			
0	22,464 (88.9%)	5,213 (91.2%)	<0.001
1	2,807 (11.1%)	504 (8.8%)	
Neurological disease			
0	24,156 (95.6%)	5,504 (96.3%)	0.021
1	1,115 (4.4%)	213 (3.7%)	
Liver disease			
0	25,068 (99.2%)	5,666 (99.1%)	0.501
1	203 (0.8%)	51 (0.9%)	
Renal disease			
0	24,409 (96.6%)	5,514 (96.4%)	0.600
1	862 (3.4%)	203 (3.6%)	
Metabolic disease			
0	21,727 (86.0%)	4,874 (85.3%)	0.158
1	3,544 (14.0%)	843 (14.7%)	
Anemia			
0	21,194 (83.9%)	4,786 (83.7%)	0.778
1	4,077 (16.1%)	931 (16.3%)	
Infectious disease			
0	25,093 (99.3%)	5,698 (99.7%)	0.001
1	178 (0.7%)	19 (0.3%)	
Hematologic diseases			
0	23,820 (94.3%)	5,588 (97.7%)	<0.001
1	1,451 (5.7%)	129 (2.3%)	
Other disease			
0	13,699 (54.2%)	3,504 (61.3%)	<0.001
1	11,572 (45.8%)	2,213 (38.7%)	
Other treatment			
0	22,477 (88.9%)	5,157 (90.2%)	0.006
1	2,794 (11.1%)	560 (9.8%)	
Charlson score			
0	7,989 (31.6%)	2,200 (38.5%)	<0.001
1	2,684 (10.6%)	637 (11.1%)	
2	2,581 (10.2%)	656 (11.5%)	
≥3	12,017 (47.6%)	2,224 (38.9%)	
sex			
Female	9,251 (36.6%)	2,608 (45.6%)	<0.001
Male	16,020 (63.4%)	3,109 (54.4%)	
Age (years)	66.243 (8.545)	67.668 (8.162)	<0.001
BMI (kg/m2)	25.477 (4.278)	26.819 (5.233)	<0.001
FEV	73.979 (19.626)	79.424 (15.435)	<0.001

OMS =0			
NO	14,886 (58.9%)	2,221 (38.8%)	<0.001
Yes	10,385 (41.1%)	3,496 (61.2%)	
Adenocarcinoma			
No	9,297 (36.8%)	1,297 (22.7%)	<0.001
Yes	15,974 (63.2%)	4,420 (77.3%)	
T			
1	9,673 (38.3%)	4,082 (71.4%)	<0.001
2	12,647 (50.0%)	1,047 (18.3%)	
3	2,951 (11.7%)	588 (10.3%)	
N			
0	19,487 (77.1%)	4,848 (84.8%)	<0.001
1	2,486 (9.8%)	580 (10.1%)	
2	3,298 (13.1%)	289 (5.1%)	
Pulmonary resection			
Limited	5,332 (21.1%)	140 (2.4%)	<0.001
Lobectomy	16,436 (65.0%)	5,555 (97.2%)	
Bilobectomy	1,417 (5.6%)	2 (0.0%)	
Pneumonectomy	2,086 (8.3%)	20 (0.3%)	
Extended resection			
0	20,766 (82.2%)	5,710 (99.9%)	<0.001
1	4,505 (17.8%)	7 (0.1%)	
sleeve			
0	24,113 (95.4%)	5,716 (100.0%)	<0.001
1	1,158 (4.6%)	1 (0.0%)	
Hospital volume	253.132 (256.848)	266.927 (254.188)	<0.001
Type of hospitals			
Non academic	2,809 (11.1%)	580 (10.1%)	<0.001
Private-for-profit	7,799 (30.9%)	1,444 (25.3%)	
Private-no-profit	2,518 (10.0%)	908 (15.9%)	
Academic	12,145 (48.1%)	2,785 (48.7%)	
Year			
2019	5,448 (21.6%)	176 (3.1%)	<0.001
2020	4,926 (19.5%)	654 (11.4%)	
2021	4,867 (19.3%)	1,134 (19.8%)	
2022	4,971 (19.7%)	1,364 (23.9%)	
2023	5,059 (20.0%)	2,389 (41.8%)	
90-day mortality	1,970 (7.8%)	178 (3.1%)	<0.001

Table S2. Variables used in propensity and Standardized Mean Difference (SMD) before matching and weighting

	Thoracotomy (25,271)	Robot assisted surgery (5,717)	SMD
Age	66.29 (8.70)	67.48 (8.33)	0.14
sex	0.63 (0.48)	0.54 (0.50)	0.169
Pulmonary disease	0.35 (0.48)	0.27 (0.45)	0.165
Heart disease	0.18 (0.38)	0.15 (0.36)	0.075
Peripheral vascular disease	0.11 (0.31)	0.09 (0.28)	0.077
Neurological disease	0.04 (0.21)	0.04 (0.19)	0.033
Liver disease	0.01 (0.09)	0.01 (0.09)	0.01
Renal disease	0.03 (0.18)	0.04 (0.19)	0.008
Metabolic disease	0.14 (0.35)	0.15 (0.35)	0.018
Anemia	0.16 (0.37)	0.16 (0.37)	0.005
Infectious disease	0.01 (0.08)	0.00 (0.06)	0.048
Hematological disease	0.06 (0.23)	0.02 (0.15)	0.179
Other disease	0.46 (0.50)	0.39 (0.49)	0.146
Other treatment	0.11 (0.31)	0.10 (0.30)	0.044
charlson score			0.179
Extended resection	0.18 (0.38)	0.00 (0.03)	0.651
sleeve	0.05 (0.21)	0.00 (0.01)	0.308
Pulmonary resection			0.905
BMI	22.52 (4.82)	12.49 (3.56)	2.368
FEV	35.69 (9.8)	39.61 (14)	0.33
OMS = 0	0.24 (0.43)	0.20 (0.40)	0.104
Adenocarcinoma	0.47 (0.50)	0.44 (0.50)	0.049
pT1	0.44 (0.50)	0.32 (0.47)	0.248
pT2	0.08 (0.27)	0.10 (0.30)	0.079
pT3	0.09 (0.29)	0.10 (0.30)	0.025
pN0	0.56 (0.50)	0.58 (0.49)	0.031
pN1	0.15 (0.36)	0.27 (0.44)	0.304
pN2	0.20 (0.40)	0.14 (0.34)	0.182
Median			0.143

Figure S1. Modeling pipeline

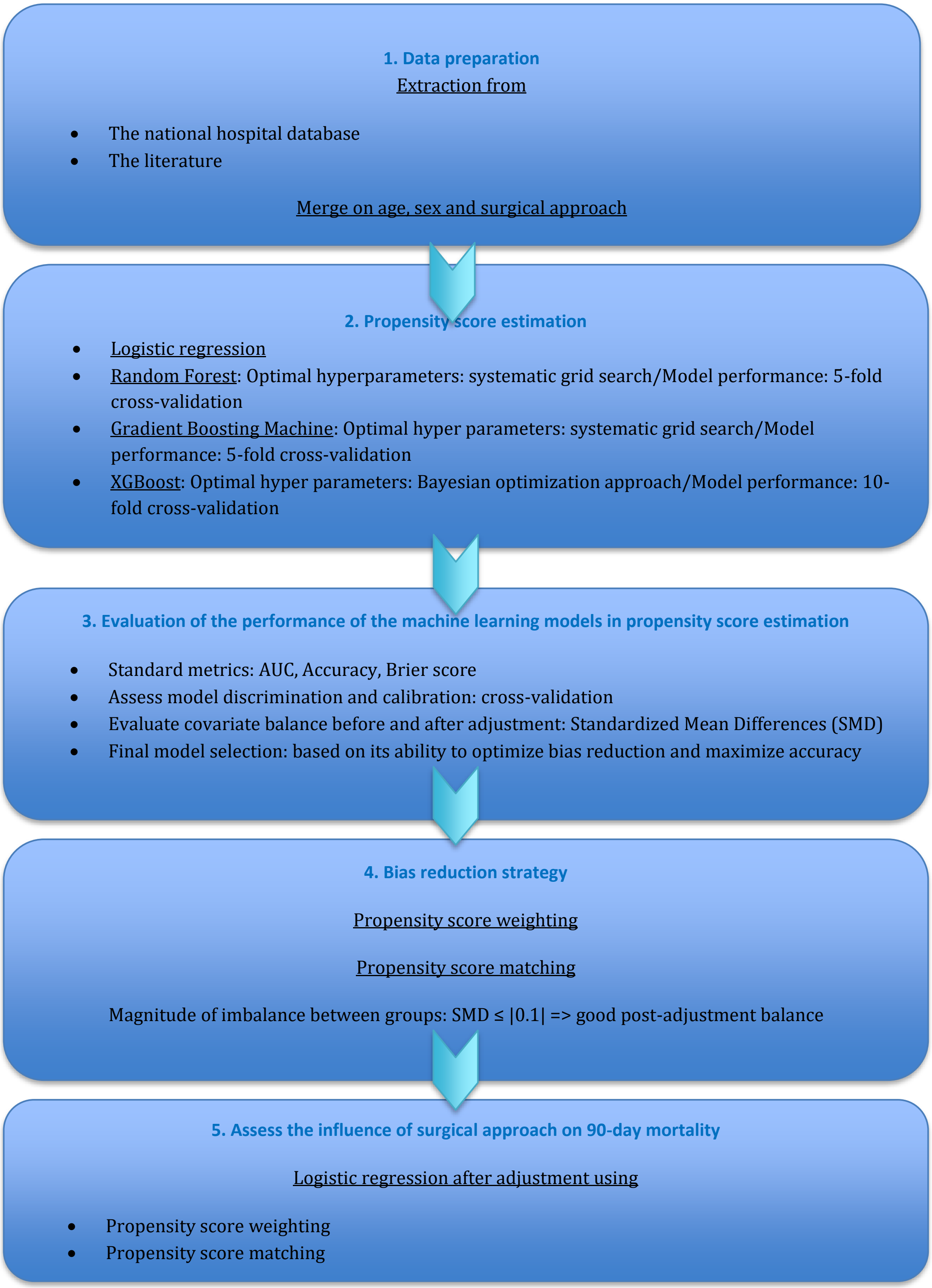

Supplement: Supplementary file 1 [file cancers-17-03347-s001.zip › cancers-3883137-supplementary.pdf]
